# The detection of flaws in austenitic welds using the decomposition of the time-reversal operator

**DOI:** 10.1098/rspa.2015.0500

**Published:** 2016-04

**Authors:** Laura J. Cunningham, Anthony J. Mulholland, Katherine M. M. Tant, Anthony Gachagan, Gerry Harvey, Colin Bird

**Affiliations:** 1 Department of Mathematics and Statistics, University of Strathclyde, Glasgow G1 1XH, UK; 2 Centre for Ultrasonic Engineering, University of Strathclyde, Glasgow G1 1XW, UK; 3 PZFlex Europe, 50 Richmond Street, Glasgow G1 1XP, UK; 4 Doosan Babcock, T&E Building, Porterfield Road, Renfrew, Glasgow PA4 8DJ, UK

**Keywords:** non-destructive testing, time reversal, ultrasonics, arrays, imaging

## Abstract

The non-destructive testing of austenitic welds using ultrasound plays an important role in the assessment of the structural integrity of safety critical structures. The internal microstructure of these welds is highly scattering and can lead to the obscuration of defects when investigated by traditional imaging algorithms. This paper proposes an alternative objective method for the detection of flaws embedded in austenitic welds based on the singular value decomposition of the time-frequency domain response matrices. The distribution of the singular values is examined in the cases where a flaw exists and where there is no flaw present. A lower threshold on the singular values, specific to austenitic welds, is derived which, when exceeded, indicates the presence of a flaw. The detection criterion is successfully implemented on both synthetic and experimental data. The datasets arising from welds containing a flaw are further interrogated using the decomposition of the time-reversal operator (DORT) method and the total focusing method (TFM), and it is shown that images constructed via the DORT algorithm typically exhibit a higher signal-to-noise ratio than those constructed by the TFM algorithm.

## 1. Introduction

The non-destructive testing of austenitic welds using ultrasound is vital for the assessment of safety critical structures such as those found in the aerospace and nuclear industries [1]. The polycrystalline microstructure of these welds is highly scattering making it difficult to detect and characterize internal defects [2–6]. To help overcome these difficulties, the use of ultrasound transducer arrays and the associated full matrix capture (FMC) data is becoming more widespread. FMC data are the complete set of *N*×*N* signals generated when each of the *N* array elements transmits in turn and the others record the scattered time domain signals. In order to characterize flaws within a structure, an image can be created by applying a delay and sum imaging algorithm to the FMC data. The total focusing method (TFM) [7–12] is such an algorithm which uses the time domain signals from the FMC dataset to create an image of the inspection area by systematically focusing on each point in the imaging domain. Another branch of imaging techniques are those which use time-reversal principles [13–21]. These methods are based on the principal of last in-first out; the delay laws from the received signal are reversed and another ultrasonic wave is sent into the material using these laws to improve focusing of the defect. It has been shown that this process can be used for selective focusing and to iteratively focus on multiple scatterers within a medium. The decomposition of the time-reversal operator (DORT) method [18,22,23] uses the singular value decomposition (SVD) of time-frequency domain data extracted from FMC data. An image of a scatterer in an inhomogeneous medium can be generated by using the eigenvectors of the response matrices which contain the phase laws that need to be applied in order to focus on the scatterer [24–27]. The method has been successfully applied in polycrystalline materials in [3,19,28], and it was shown that the DORT technique could successfully differentiate the contribution of the defect from noise arising from the microstructure. More recent work by Marengo *et al.* [29,30] explores detection (using acoustic, electromagnetic or optical data) of unknown scatterers embedded in unknown complex background media using the time-reversal mirror.

A common factor with the classical delay and sum imaging algorithms discussed above is the requirement of a subjectively chosen imaging threshold. Previous work has been carried out in [31–33] to address this issue and size cracks objectively in the time-frequency domain. In this paper, an objective detection criterion specific to austenitic welds is proposed, based on the SVD of the response matrix. The distribution of these singular values has already been used as an indicator of multiple scattering in coarse-grained media [34]. To illustrate, the method is applied to data arising from both finite-element simulations and experiments. Having detected a defect, the DORT imaging method is then used to image a crack within an austenitic weld and the results are compared with those produced using the TFM.

## 2. The decomposition of the time-reversal operator method

The DORT method [18] is a detection technique which uses the SVD of FMC data to determine the time-reversal invariants. The method can be divided into two stages: the first stage determines the existence of a defect and the second stage concerns the imaging of the defect and its localization within the structure.

To begin, the DORT method transforms the time domain FMC data, *H*_*N*×*N*×*N*_T__={*H*_*ijp*_:*i*,*j*=1,…,*N*, *p*=1,…,*N*_T_} (where *N* is the number of phased array elements and *N*_T_ is the number of time samples taken), into the time-frequency domain via a time-windowed discrete Fourier transform (DFT). For each time, *T*_*p*_, and time window, Δ*T*, a submatrix of *H* is given by

2.1 $${\hat{H}}_{i,j}(T_{p},t)={\hat{H}}_{i,j}(t-T_{p})W(t)\;i,j=1,\ldots ,N$$

where

2.2 $$\begin{array}{rl} W(t) & =\left\{ \begin{array}{ll} 1, & \text{if }t\in \left[\frac{-\Delta T}{2},\frac{\Delta T}{2}\right],\\ 0, & \text{otherwise}, \end{array}\right.\\ \text{and}\;T_{p} & =t_{1}+\Delta t(p-1). \end{array}$$

Here, *t*_1_ is the start time of the signal being sampled (in practice, this will be large enough to not include reflections from the front face of the structure being inspected). The DFT is calculated to produce the set of response matrices, *K*_*N*×*N*_(*T*_*p*_,*f*_*q*_), at fixed time *T*_*p*_ (with *p*=1,…,*N*_T_) and fixed frequencies *f*_*q*_=*q*/Δ*t* (with *q*=1,…,*N*_f_, where *N*_f_ is the total number of frequency samples). The SVD of the response matrix *K*(*T*_*p*_, *f*_*q*_) for each fixed time, *T*_*p*_, and frequency, *f*_*q*_, pair is then calculated using

2.3 $$K=U\Lambda V^{*},$$

where *Λ* is a diagonal matrix containing real, positive singular values λ_*k*_, *k*=1,…,*N*, the columns of *U* are the left singular vectors and the rows of *V* are the right singular vectors. For isotropic scatterers, each singular value is associated with one scatterer in the material. In the case of small, non-isotropic scatterers (where the diameter of the scatterer is much smaller than the wavelength), four singular values are associated with the scatterer (the largest is associated with the spherically symmetric part of the scattering amplitude and the other three are associated with the directional part). Where the scatterer is larger than the wavelength, there exist many singular values associated with it. Once the SVD for each time-frequency pair, (*T*_*p*_,*f*_*q*_) is determined, the largest singular value, λ_1_(*T*_*p*_, *f*_*q*_), is normalized using the quadratic mean of all the singular values at that time-frequency pair [35]

2.4 $${\bar{\lambda }}_{1}(T_{p},f_{q})=\frac{\lambda_{1}(T_{p},f_{q})}{\sqrt{\left(1/N)\sum_{p=1}^{N}\lambda_{p}^{2}(T_{p},f_{q}\right)}}.$$

If there is a flaw present, the normalized first singular value will be above a threshold value, *τ*. This stage can be used for objective flaw detection where no *a priori* knowledge of the material being inspected is required. In this paper, a detection threshold, *τ*, specific to austenitic welds is calculated.

The second stage in the DORT method concerns image reconstruction and requires the input of a homogenized material wave speed, *c*. The image is generated using back propagation, where the propagation operator is a time harmonic spherical wave (Green's function), and the focusing is determined by the right eigenvectors, *V*_1_(*T*_*p*_,*f*_*q*_), associated with the singular values where ${\bar{\lambda }}_{1}(T_{p},f_{q})>\tau$. The image domain is discretized by a grid, where the number of pixels in the vertical direction of this grid is dictated by the number of time samples (*N*_T_) and the number of pixels along the horizontal axis is a free parameter which will be denoted by ${\hat{x}}_{l}$ (*l*=1,…,*N*_*L*_). So, for a fixed point in the image space $\left({\hat{x}}_{l},z_{p}\right)$, the propagation operator is discretized into a 1×*N* vector *G*_*lp*_; the elements of which are given by

2.5 $$g_{jlp}(\;f_{q})=\frac{e^{i2\pi f_{q}r_{jlp}/c}}{\sqrt{r_{jlp}}}\;j=1,\ldots ,N$$

where

2.6 $$r_{jlp}=\sqrt{z_{p}^{2}+{\left(x_{j}-{\hat{x}}_{l}\right)}^{2}},$$

*z*_*p*_=*cT*_*p*_/2 is the depth in the material and *x*_*j*_ is the spatial position of array element *j*. Each value in the image, $I(T_{p},{\hat{x}}_{l})$, is calculated using the absolute value of the back propagated wave which is focused using the right eigenvector associated with the largest singular values that lie above the threshold *τ*. Hence,

2.7 $$I(T_{p},{\hat{x}}_{l})=\sum_{f_{q}|\bar{\lambda }>\tau }\lambda_{1}(T_{p},f_{q})|V_{1}(T_{p},f_{q})G_{lp}^{*}|,$$

where $G_{lp}^{*}$ is the complex conjugate of Green's function vector signifying the time-reversed stage.

## 3. Detection of flaws in austenitic welds

The first stage of the DORT method investigated here uses the distribution of the largest singular values, ${\left\{{\bar{\lambda }}_{1}\right\}}_{m}$, *m*=1,…,*N*_T_*N*_f_, from the time-frequency response matrices, *K*_*ij*_(*T*_*p*_,*f*_*q*_). If the weld contains a defect this distribution will give rise to values which are significantly larger than a specified detection threshold, *τ*. In this paper, a detection threshold specific to an austenitic weld is empirically calculated using a finite-element simulation. The configuration in this work differs from the multiple scattering regime in [36], as multiple scattering does not dominate the received signal here. The problems which arise are mainly due to the weld's grain structure, which causes the wave to scatter and refract as it passes through the weld.

### (a) Finite-element simulated data

**Figure 1.**
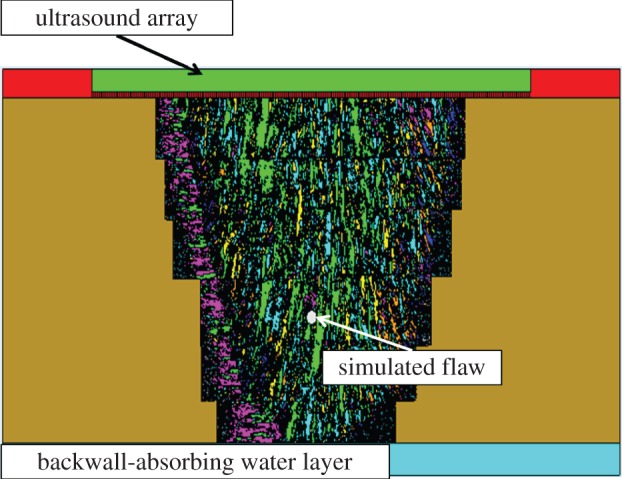
Internal microstructure of an austenitic weld as input into the finite-element model implemented in the software package PZFlex. The weld microstructure is surrounded by stainless steel and backed by water. The different colours signify the grain structure in the material, where each colour represents a particular grain orientation [37].

In order to run accurate finite-element simulations of waves propagating through an austenitic weld, it is imperative to have knowledge of the internal microstructure of the weld as the anisotropic nature of the material has a marked effect on the passage of elastic energy through it. A simulation in the software package PZFlex which included the microstructure of an austenitic weld was generated in [37], where considerable effort was expended in fully characterizing the weld microstructure using Electron Backscatter Diffraction (EBSD) measurements taken from [5]. Although the weld comprises a single anisotropic material, grain boundaries are present within the weld; a consequence of the welding process. In fact, the internal microstructure can be viewed as a partitioning of the weld area into a large set of sub-regions, each one with an assigned crystal orientation. Within the finite-element simulation, the internal geometry is meshed with elements with dimension equal to λ/15 (where λ is the wavelength), approximately 200 *μ*m in this case, which is below the Rayleigh scatterer limit of 300 μm and sufficient to model accurate wave propagation [37]. Each element is assigned a crystal stiffness and orientation, and groups of contiguous elements with the same stiffness and orientation form a grain within the weld. For this work, new FMC datasets were generated where a zero volume crack and side drilled holes of varying radii were inserted into this anisotropic geometry within the PZFlex software. The theoretical focusing width (equivalent to the lateral resolution) of the phased array transducer coupled with the sample geometry modelled in the simulation is approximately 3 mm according to the Rayleigh criterion [38] and the series of flaws inserted into the simulation represent cases where the flaw is less than (a 1 mm diameter side drilled hole), commensurate with (a 2.5 mm side-drilled hole) and larger than (a 5 mm long crack) this measurement of spatial resolution. A schematic demonstrating the set up is shown in figure 1. A square grid was used within the simulation and so the crack is represented by a thin rectangular void (no stiffness) and behaves as a perfect reflector. The simulation also included a 64 element ultrasonic array (the parameters of which are given in table 1) placed directly above the weld microstructure. A 1.5 MHz single cycle sinusoid was transmitted by one element and the time domain echo received by all 64 elements was recorded. The transmitting element was then systematically changed by moving along the array until the full matrix of time domain data was captured, for a total of 64 unique simulations per virtual inspection scenario. By applying the TFM algorithm to the collected FMC data, the known location of the back wall was used to estimate a constant longitudinal wave speed (the finite-element simulation does include both longitudinal and shear waves but only the longitudinal wave speed was deemed necessary as it is associated with the propagation of the largest amplitude part of the wave front). The RMS longitudinal velocity was calculated as 5758 m s^−1^, with a standard deviation of 146.2 m s^−1^ (these were calculated using the known distances and corresponding times at which the echo from the back wall occurred in the A-scans where the transmission and reception took place on the same element). In the 1.5 MHz inspection simulated, the correlation length [39] was estimated as approximately λ/8, (where λ is the wavelength). This simulated data and its associated parameters are used in all forthcoming sections to test the methods proposed in this paper.

**Table 1.** Parameters used in the finite-element simulations of ultrasonic waves probing an austenitic weld. The array and material parameters in each simulation remained constant and only the type and size of the flaw was varied.

phased array inspection parameters	value (units)
number of elements	64 (–)
pitch	2 (mm)
transducer centre frequency	1.5 (MHz)
estimated longitudinal wave speed in host material	5800 (m s^−1^)
density of host material	7874 (kg m^−3^)
flaw diameter (side drilled holes)	0.5, 1.25 (mm)
flaw length (crack)	5 (mm)
depth of flaw	50 (mm)
depth of sample	78.6 (mm)
time sample rate	17.3 (ns)

### (b) A comparison of the singular value distribution from an austenitic weld to the quarter circle law

In this section, the distribution of singular values from the response matrix *K*_*ij*_(*T*_*p*_, *f*_*q*_) arising from a finite-element simulation of ultrasonic waves in an austenitic weld is calculated. The data arising from the simulated inspection of an austenitic weld containing *no flaw* are used to establish an empirical understanding of the singular value distribution for this material. The parameters used to generate the inter-element response matrix are given in table 2. The singular values, λ_*m*_(*T*_*p*_,*f*_*q*_) (where *m*=1,…,*N*_T_*N*_f_), are normalized using equation (2.4). These singular values are then segregated into bins to produce a probability density distribution. The bin intervals are given by

3.1 $$b_{s}=[(s-1)B,sB],$$

where *B* is the bin width and *s*=1,…,*N*_*s*_ (*N*_*s*_ is the total number of bins). The total number of singular values contained within each bin is denoted as *D*(*b*_*s*_). The probability density distribution of the singular values is estimated [35] by

3.2 $$\rho (b_{s})=\frac{D(b_{s})}{nB},$$

where *n*=*N*×*N*_T_×*N*_f_ is the total number of normalized singular values arising from all of the response matrices *K*_*ij*_(*T*_*p*_,*f*_*q*_), where *p*=1,…,*N*_T_ and *q*=1,…,*N*_f_. This distribution is compared to the quarter circle law (QCL) [40] which is given by

**Figure 2.**
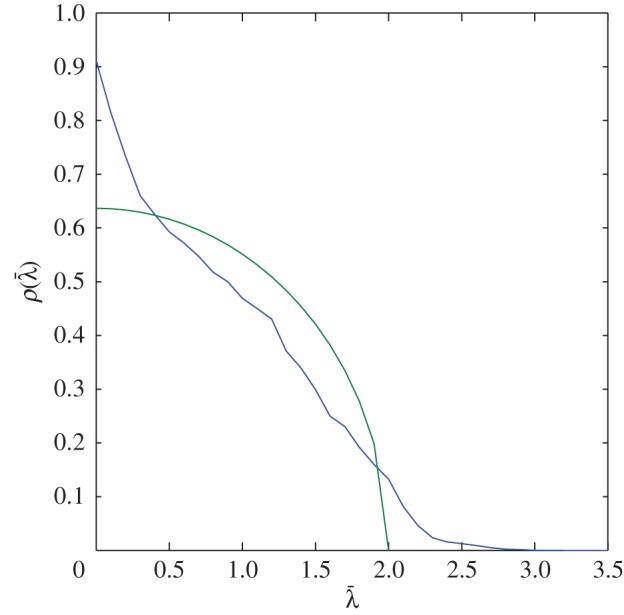
This plot shows the distribution of normalized singular values (given by equation (3.2), blue line), as calculated from the response matrices, *K*_*ij*_(*T*_*p*_,*f*_*q*_), arising from a finite-element simulation of an austenitic weld. The parameters used in this simulation are given in table 1. This distribution is compared with that given by the QCL (given by equation (3.3), green line) which is derived from RMT.

3.3 $$\rho_{QC}(\bar{\lambda })=\frac{\sqrt{4-{\bar{\lambda }}^{2}}}{\pi },\;\mathrm{where}\;0<\bar{\lambda }<2$$

and gives the distribution of singular values from a square random matrix derived from random matrix theory (RMT). The entries of the random matrix have to be independently and identically distributed for this law to be applied. Figure 2 shows the comparison between the QCL (green line) given by equation (3.3) and the distribution, *ρ*(*b*_*s*_) of singular values from the response matrices *K*_*ij*_(*T*_*p*_,*f*_*q*_) (see equation (3.2) and figure 2, blue line) arising from the finite-element simulated data of an austenitic weld. It can be seen from this plot that the distribution of singular values from the austenitic weld does not fit with the QCL. The overall shape of the distribution is not dissimilar to that shown by Shahjahan *et al.* [34] and is thus characteristic of multiple scattering in coarse-grained media. As no flaw is contained in these simulations, these large singular values must stem from scattered waves emanating from some of the larger grains in the weld. These are not to be classified as flaws and so the detection criterion based on RMT and the QCL is not suitable for inspecting austenitic welds. The next subsection investigates this in more depth and arrives at a detection criterion for defects in austenitic welds using the distribution of the largest singular values from the response matrices *K*_*ij*_(*T*_*p*_,*f*_*q*_) (*p*=1,…,*N*_T_ and *q*=1,…,*N*_f_).

**Table 2.** Parameters used to generate the inter-element response matrix, *K*_*ij*_(*T*_*p*_,*f*_*q*_), from the ultrasonic data arising from the finite-element simulation of a phased array inspection of an austenitic weld containing a series of flaws.

parameters to create inter-element response	value
time window (Δ*T*)	4.5 μs (14 mm)
number of time windows (*N*_T_)	140
number of frequencies (*N*_f_)	32
time step (Δ*t*)	159 ns (0.5 mm)

### (c) A threshold for detection of flaws in austenitic welds using the largest singular value

**Figure 3.**
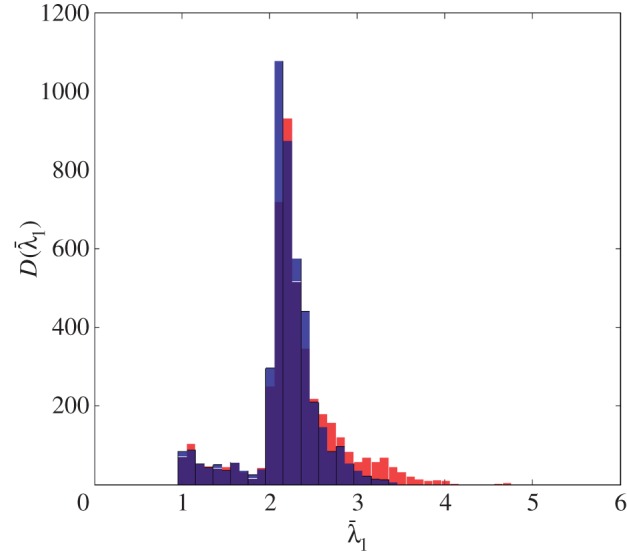
This plot shows the histogram $D({\bar{\lambda }}_{1})$ of the largest singular values across all time-frequency pairs ( *p*=1,…,*N*_T_, *q*=1,…,*N*_f_) calculated from the response matrix, *K*_*ij*_(*T*_*p*_,*f*_*q*_), associated with the finite-element simulated data of an austenitic weld (*a*) without a defect (blue bars) and (*b*) with 1.25 mm radius side drilled hole inclusion (red bars). The dark blue areas represent where the two lie on top of each other.

**Figure 4.**
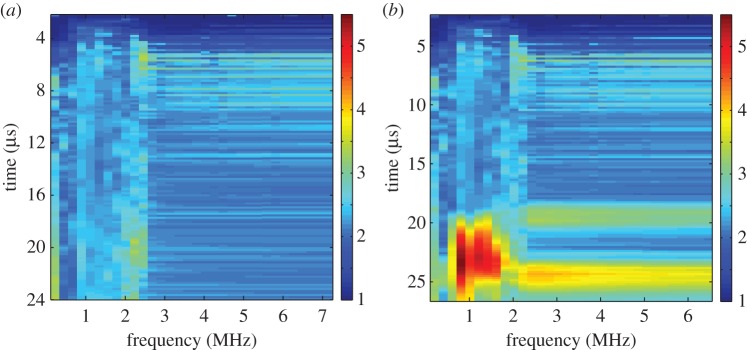
Heat maps of the largest singular values ${\bar{\lambda }}_{1}(T_{p},f_{q})$ arising from the response matrix *K*_*ij*_(*T*_*p*_,*f*_*q*_) (table 2), calculated from the finite-element simulated data of ultrasonic waves propagating through a heterogeneous austenitic weld, where (*a*) no defect is included and (*b*) a 1.25 mm radius side drilled hole is included within the weld geometry.

The distribution of the largest singular values of the response matrices, *K*_*ij*_(*T*_*p*_,*f*_*q*_), from the finite-element simulated data of an austenitic weld (as outlined in §3a), with and without a flaw inclusion, are analysed in this subsection. The aim is to determine a threshold specific to austenitic welds which can be used as an objective flaw detection method. Histograms of the normalized largest singular values for all time-frequency pairs calculated from the response matrix, *K*_*ij*_(*T*_*p*_,*f*_*q*_), from the finite-element simulated data of an austenitic weld containing no flaw (blue bars) and with a 1.25 mm radius side drilled hole flaw (red bars) are shown in figure 3. This figure shows that when there is no flaw included within the austenitic weld (blue bars) the highest concentration of largest singular values lie between 2 and 2.5, with the largest being 3.7. When the flaw (a 1.25 mm radius side-drilled hole) is included (red bars), there is still a large proportion of the first singular values lying between 2 and 2.5; this is to be expected as these correspond to the scattering arising from the grains within the weld structure. However, it is also clear that a significant proportion of the first singular values are greater than 3.7. These must stem from backscatter by the flaw. It is concluded from this figure that there is a notable difference between the distribution of first singular values from the response matrices when there is a flaw in the austenitic weld and from that when there is no flaw present. The distribution of the largest singular values can also be viewed as a heat map in the time-frequency domain as shown in figure 4. The distribution where a 1.25 mm radius side drilled hole flaw was included in the finite-element simulation is shown in figure 4*b*, and it is clear that there is a cluster of large first singular values between 0.5–1.5 MHz and 20–25 μs. By visually comparing this distribution to that shown in figure 4*a*, where there is no defect, it is clear that the inclusion of a defect gives rise to significantly larger singular values. In this work, the detection threshold is taken to be *τ*=3.7, this is the largest singular value from all of the response matrices where no defect is included within the finite-element simulation (as shown by the blue bars in the histogram in figure 3).

### (d) Results when the detection method is applied to finite-element simulated data

**Figure 5.**
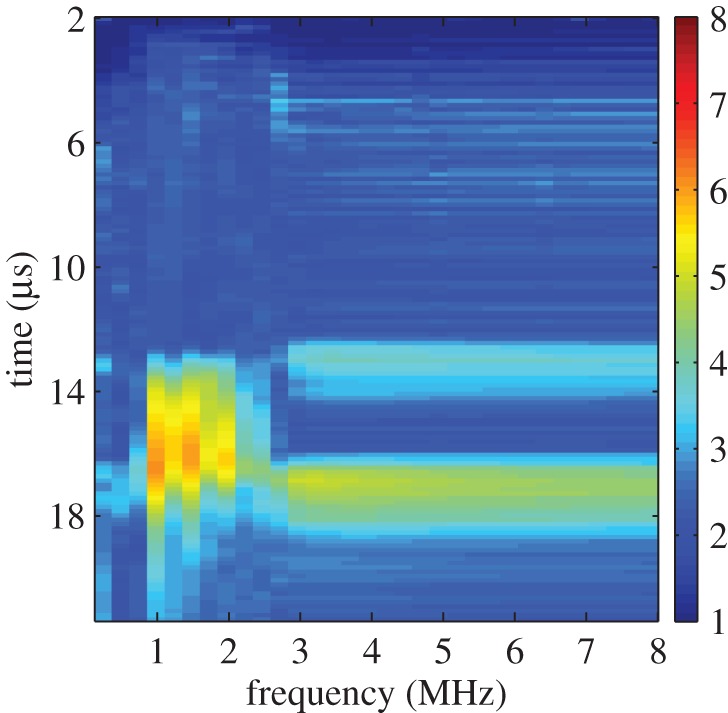
Heat map of the largest singular values ${\bar{\lambda }}_{1}(T_{p},f_{q})$ arising from the response matrix *K*_*ij*_(*T*_*p*_,*f*_*q*_) (table 2), calculated from the finite-element simulated data of ultrasonic waves propagating through a heterogeneous austenitic weld, where a crack of length 5 mm is inserted into the PZFlex simulation of ultrasonic waves propagating through an austenitic weld.

The detection algorithm summarized in §3c is applied here to other finite-element simulations. The time step (Δ*t*=159 *ns*) corresponds to a spatial step of 0.5 mm and the time window Δ*T*=4.5 μs is approximately 20% of the total time that the wave front takes to reach the back wall of the test piece. Figure 5 shows the distribution of the largest singular values across the time and frequency domain. The crack has clearly and objectively been detected as there exists a cluster of singular values larger than the threshold (*τ*=3.7). If one compares this plot with the equivalent when the weld microstructure is removed to create a homogeneous media (not shown for brevity), then it is clear that the singular values are lower here. This is due to less energy reaching the flaw (and in turn being received back by the transducer) as some of the energy in the ultrasonic waves has been scattered by the grain boundaries within the weld.

**Figure 6.**
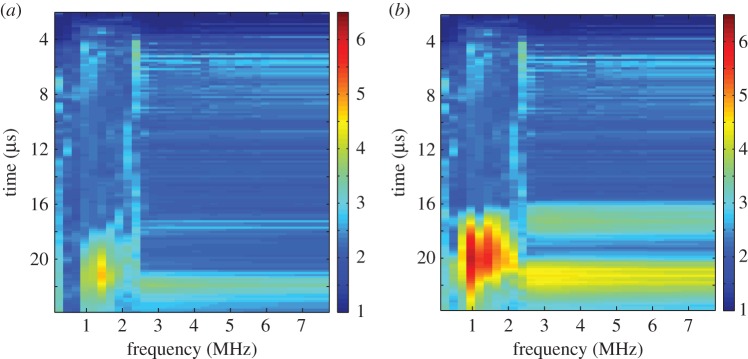
Heat maps of the largest singular values ${\bar{\lambda }}_{1}(T_{p},f_{q})$ arising from the response matrix *K*_*ij*_(*T*_*p*_,*f*_*q*_) (table 2), calculated from the finite-element simulated data of ultrasonic waves propagating through a heterogeneous austenitic weld, where side drilled holes of radii (*a*) 0.5 mm and (*b*) 2.5 mm have been embedded within the weld geometry.

Figure 6 shows the largest singular values in time-frequency space when side-drilled holes of radius (*a*) 0.5 mm and (*b*) 2.5 mm are inserted into the finite-element simulations which include the heterogeneous weld material. The parameters used to create the corresponding time-frequency response matrices are summarized in table 2. Again, it is clear from these time frequency distributions that in each case there exist singular values larger than the detection threshold and it can be concluded objectively that there exists a flaw within the structure. As expected, as the radius of the side drilled hole is increased the value of the singular values associated with the flaw also increase.

### (e) Results when the detection method is applied to experimental data

**Figure 7.**
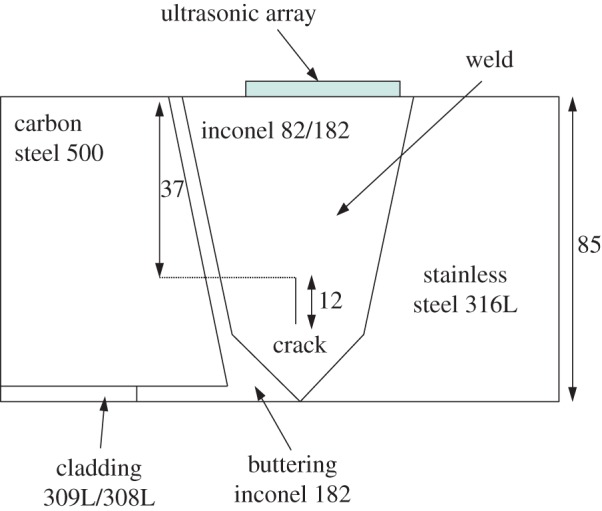
This schematic shows the test piece used to collect ultrasonic data. The block consists of a weld (the material of the weld is inconel 82/182) which joins a piece of stainless steel 316L and carbon steel 500. There is also some cladding and buttering material between the carbon steel and the weld. The test piece includes a 12 mm vertical crack 37 mm from the front face of the test piece which is 85 mm total in depth. The ultrasonic array, which has 45 elements and a central frequency of 5 MHz, was placed just to the right of the centre of the weld. (Online version in colour.)

The detection algorithm presented in §3c is now applied to experimental data from a test piece which contains an inconel 82/182 weld [5]. The parent material to the right of the weld is 316L stainless steel and to the left is carbon steel with an inconel 182 buttering layer between this and the weld. A schematic of the test piece is shown in figure 7. The inspection was carried out by a 5-MHz linear array (Vermon, Tours, France) combined with a Dynaray (Zetec, Quebec, QC, Canada) array controller (see table 3 for a summary of the array and material properties). An FMC was collected from the sample where the array was positioned off centre, directly above the weld (as shown in figure 7) so as to include scattering by the 12 mm vertical zero-volume flaw (crack) present in the centre of the weld, located 37 mm from the surface. Note that since the scenario considered is a linear phased array inspection, we are concerned with only two dimensions, and as the defect is a zero-volume crack, then it effectively only has one dimension (length). Although the crack is perpendicular to the array, a planar phased array was chosen over an oblique incidence inspection as it represents a very difficult case in which the TFM struggles to detect anything. Such a scenario could arise in practice if there was limited access to the component of interest. In order to demonstrate the effect of a flaw inclusion on the largest singular value distribution, FMC data were also collected from an area within the weld, where it was known that there was no flaw.

**Table 3.** The parameters associated with experimental data from a test piece which contains an inconel 82/182 weld [5].

ultrasonic transducer array parameters	value (units)
number of elements firing	45 (–)
pitch	0.7 (mm)
centre frequency	5 (MHz)
bandwidth (–6 dB)	60 (%)
average velocity	5780 (m s^−1^)
density of host material	8280 (kg m^−3^)
flaw	vertical crack, 12 (mm)
depth of flaw	37 (mm)
depth of sample	85 (mm)
time sampling	10 (ns)

**Figure 8.**
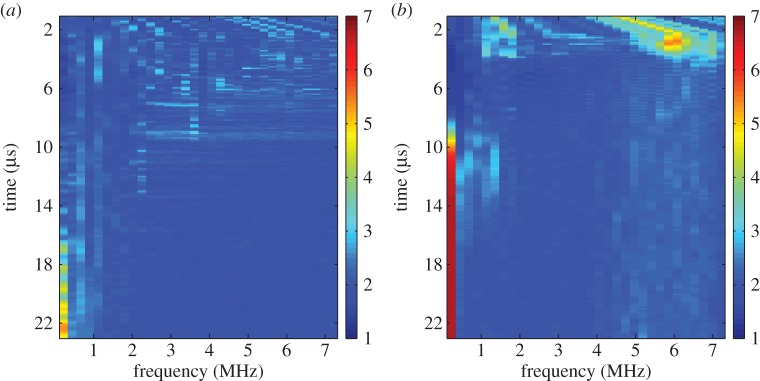
The largest singular value plots from the experimental FMC data from the austenitic weld (see figure 7 for a schematic) where (*a*) there is no flaw and (*b*) where there is a 12 mm long vertical crack.

**Figure 9.**
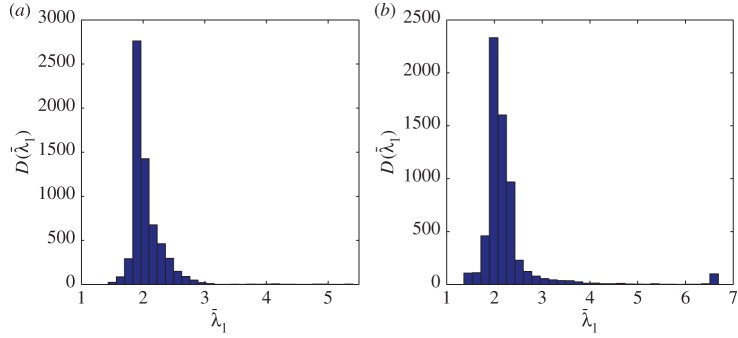
The histograms of the largest singular values across all time and frequency pairs from the experimental FMC data from an austenitic weld where (*a*) there is no flaw and (*b*) where there is a 12 mm long vertical crack. (Online version in colour.)

The time-frequency response matrix was calculated in both cases with the parameters as summarized in table 4. The normalized first singular value distributions, ${\bar{\lambda }}_{1}$ (equation (2.4)), are shown in figure 8, where (*a*) there is no defect in the inspection area and (*b*) a 12 mm long, vertical, zero-volume crack is present within the inspection area. It is clear from figure 8*b* that there are singular values larger than the detection threshold (*τ*=3.7) at the lower frequencies. These are associated with the crack and occur at the lower frequencies as the crack is long in comparison with the wavelength (the crack length to wavelength ratio is 10.4). In the no flaw case (figure 8*a*), there are some significant singular values occurring at around 22 μs which can be attributed to scattering by the back wall. The histograms corresponding to these largest singular values are shown in figure 9 and by comparing figure 9*a*,*b*, it is clear that there is a higher proportion of singular values that exceed the threshold *τ*=3.7 when a flaw is present in the inspection area. Indeed, in figure 9, there is an extremely large singular value around 6.5.

**Table 4.** Parameters used to generate the inter-element response matrix, *K*_*ij*_(*T*_*p*_,*f*_*q*_), arising from the experimental ultrasonic data summarized in table 3.

parameters to create inter-element response	value
time window (Δ*T*)	5.9 μs (17 mm)
number of time windows (*N*_T_)	200
number of frequencies (*N*_f_)	32
time step (Δ*t*)	117 ns (0.34 mm)

## 4. Imaging flaws in austenitic welds using the decomposition of the time-reversal operator and total focusing methods

In this section, the imaging stage of the DORT algorithm is applied to data arising from both the finite-element simulation and experimental inspection of an austenitic weld with defects. In addition, the TFM is applied to these FMC datasets to produce images for comparison. An image is created using the DORT method once the flaw has been detected using the largest singular value distribution. This highlights the time-frequency pairs where the corresponding eigenvectors can be used to back-propagate and create an image of the defect (see equation (2.7)). It is important to note that within this work the most basic form of TFM has been used to generate the forthcoming images [10] and that there are more advanced versions of the method available [8,7]. In the following sections, all the images have been plotted on a decibel scale $I_{\mathrm{dB}}=20\log_{10}(I/I_{\max})$, where *I* is the image matrix produced and $I_{\max}$ is its maximum.

### (a) Image reconstruction of flaws within an austenitic weld using finite-element simulated data

In this section, the DORT method and the TFM are applied to the data arising from the finite-element simulation of the phased array inspection of an austenitic weld incorporating a side drilled hole and a horizontal crack. The images from the DORT method can be compared with those generated using the TFM via the signal-to-noise ratio (SNR). In this work, the SNR is calculated by $\text{SNR}=20\log_{10}(A_{\max}/A_{0})$, where $A_{\max}$ is the maximum amplitude in the image and *A*_0_ is the maximum amplitude from a region in the image which does not contain any scattering from the flaw but does contain noise. Note that an aspect of subjectivity arises from the choice in the noisy region from which *A*_0_ is determined.

**Figure 10.**
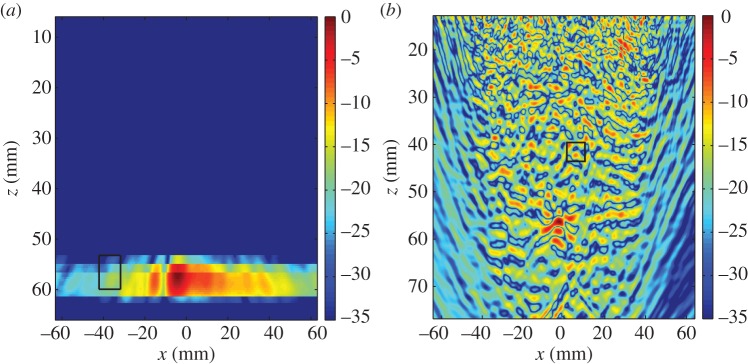
The image arising from the simulation of a side drilled hole with radius 0.5 mm embedded in an austenitic weld within PZFlex, reconstructed using (*a*) the DORT method and (*b*) TFM. The black rectangle shows the region used for the SNR calculation.

The imaging methods are first applied to data arising from the finite-element simulation of the inspection incorporating a 0.5 mm radius side drilled hole within the weld microstructure. The resulting image using the DORT algorithm is shown in figure 10*a*. From this image, it can be seen that the DORT method has successfully found the side drilled hole but its shape and size are not recovered. The location of the imaged side drilled hole is out by approximately 4 mm in the horizontal direction and 3 mm in the vertical direction (using the maximum amplitude of the point spread function from the image as a reference point). The SNR of this image is 23 dB, calculated using the noisy region enclosed by the black box. The TFM was also applied to this dataset to produce the image in figure 10*b* which has been cropped to reduce the effects of the front face and back wall reflections. It can be observed that the scattering from the flaw is close to the order of the noise, and it is difficult to find and identify the flaw in this clutter. The SNR in this image is calculated using the maximum amplitude in the region enclosed by the black box to give a measurement of 8 dB. In this case, the DORT has proved superior in terms of detection and demonstrates a marked advantage over the TFM in the detection of a sub-wavelength defect embedded in a noisy host medium.

**Figure 11.**
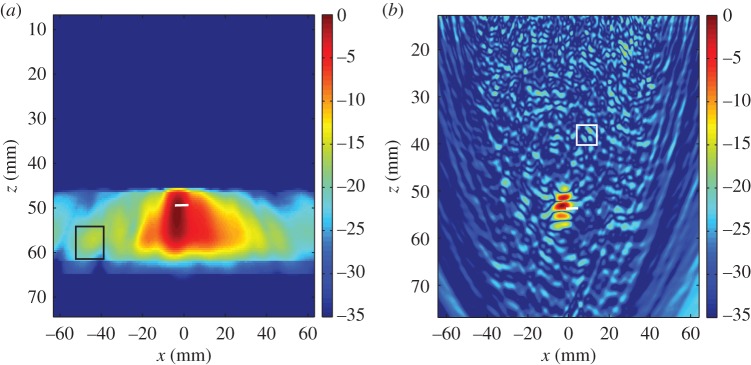
The image of a 5 mm horizontal crack in an austenitic weld from finite-element-simulated FMC data generated using (*a*) the DORT method and (*b*) TFM. The white line indicates the true location and length of the crack. The black rectangle shows the region used for the SNR calculation.

The next configuration considered is when a horizontal crack of length 5 mm is included within the simulation (see table 1 for the relevant parameters). The image reconstructed using the DORT method is shown in figure 11*a*, where the white line demonstrates the actual location and length of the flaw. The SNR in figure 11 is 19 dB, where *A*_0_ is taken to be the maximum amplitude in the region depicted by the black rectangle. The TFM was subsequently applied to these data to generate the image shown in figure 11*b* where again, the white line indicates the true location and length of the crack. The SNR from the TFM image is 20 dB, where the estimate of noise was calculated within the region enclosed by the white rectangle. In this particular case, the TFM proves to be superior to the DORT algorithm. As discussed earlier, it has been shown that many eigenvalues can be associated with one scatterer depending on its size and characteristics [41], and it is demonstrated here that failure to account for all the relevant singular values means that the DORT algorithm is unable to characterize the nature and size of the flaw. A potential avenue for future work could entail further investigation of the singular value distribution when there is a crack-like defect present and subsequently developing the DORT method to include more than just the largest singular value in the image reconstruction.

### (b) Image reconstruction of a flaw within an austenitic weld using experimental data

**Figure 12.**
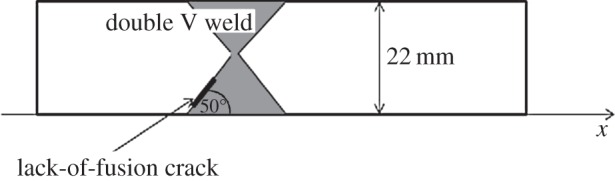
This schematic shows the test sample used to collect the experimental data as summarized in table 5. A 6 mm lack-of-fusion crack orientated at 50° with respect to the *x*-axis is present on the boundary of the double V weld between two austenitic plates of 22 mm depth.

In this section, the DORT and TFM imaging algorithms are applied to a second experimental FMC dataset. The test sample under inspection was manufactured from 316L stainless steel and constructed from two welded austenitic plates. The defect of interest was a lack-of-fusion crack tilted at 50° with respect to the *x*-axis, with 6 mm height (approx. 7.8 mm length). The lower crack tip begins 2 mm from the backwall. A 128 element linear array (Vermon, Tours, France) with a 5 MHz centre frequency was used to carry out the inspection, combined with the Dynaray (Zetec, Quebec, Canada) array controller. The array was placed directly above the weld (figure 12) which measured 22 mm in depth. The experimental parameters are listed in table 5 and the parameters used to generate the time-frequency response matrix can be found in table 6.

**Table 5.** The parameters associated with experimental data from a test piece which contains an inconel 82/182 weld [5].

ultrasonic transducer array parameters	value (units)
number of elements firing	128 (–)
pitch	0.7 (mm)
centre frequency	5 (MHz)
bandwidth (–6 dB)	60 (%)
time sampling	10 (ns)

**Table 6.** Parameters used to generate the inter-element response matrix, *K*_*ij*_(*T*_*p*_,*f*_*q*_), arising from the experimental ultrasonic data summarized in table 5.

parameters to create inter-element response	value
time window (Δ*T*)	5.9 μs (17 mm)
number of time windows (*N*_T_)	200
number of frequencies (*N*_f_)	32
time step (Δ*t*)	117 ns (0.34 mm)

**Figure 13.**
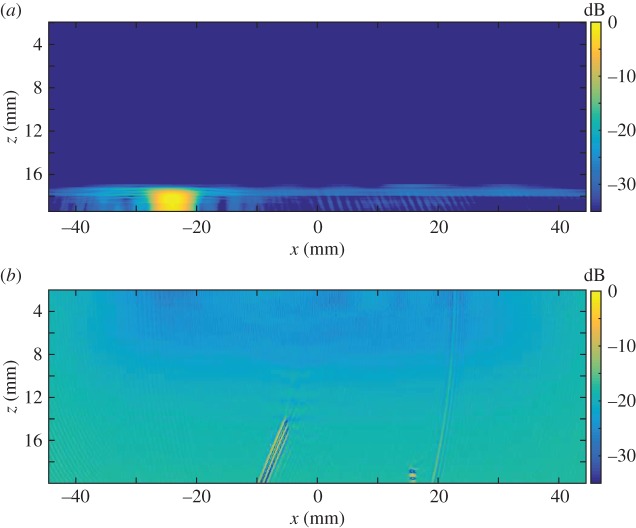
Images of a 6 mm lack-of-fusion crack at a 50° tilt (with respect to the *x*-axis), arising from experimental FMC data (as described in table 5) generated using (*a*) the DORT method and (*b*) TFM.

The DORT method was applied to this FMC dataset to produce the image shown in figure 13*a*. A high amplitude region attributed to the flaw is reconstructed at a depth of approximately 17 mm (compared to the known position of the upper crack tip at 14 mm). The predicted location of the flaw along the horizontal axis is shifted to the left from the known location as shown in the corresponding TFM image (figure 13*b*). It can be surmised that this is caused by the tilted angle of the crack; the strongest scattering is received by elements to the left of the flaw and it is only this information that the DORT algorithm exploits. Although the crack-like nature of the defect is not recovered using the DORT algorithm (this information is not fully captured using only the largest singular value), the indication that a flaw exists is undeniable and an impressive SNR of 47 dB is achieved. For comparison purposes, a TFM image of the flaw was constructed using the experimentally derived pressure wave speed of 5820 m s^−1^. The overall location and characterization of the flaw is improved in this image, however a less impressive SNR of only 16.8 dB is achieved. The higher SNR evident in the image constructed by the DORT algorithm also suggests that it has improved detection capabilities in coarse-grained media over the standard TFM and indeed, further evidence to support this conclusion is shown in [42]. The overall conclusion is that due to the limited information used by the DORT algorithm, characterization proves problematic. However, for detection purposes the DORT method presents a robust alternative to the TFM which can sometimes fail in cluttered media.

## 5. Conclusion

In this paper a flaw detection algorithm, based on the first stage of the DORT method was outlined, within which a detection criterion specific to austenitic welds was proposed. This detection algorithm was then applied successfully to data arising from a finite-element simulation of the phased array inspection of an austenitic weld containing a side drilled hole (of radius 0.5 mm) and a horizontal crack (of length 5 mm). In addition, the method was successfully applied to experimental FMC data from an austenitic weld with a 12 mm, vertical zero-volume crack. In the latter part of this paper, the DORT algorithm was used to image the flaws. The data arising from the simulated inspection of a 0.5 mm side drilled hole was interrogated by both the DORT method and the classical TFM imaging technique. In this case, the DORT showed an improved detection capability over the TFM in which it was difficult to separate the defect from background noise. However, on examination of the simulated data incorporating a 5 mm crack parallel to the array, the TFM exhibited its superior characterization abilities. This was not surprising as the data exploited by the DORT (the largest singular values of the time-frequency response matrices) does not contain information on the nature of the defect. To improve upon this aspect of the algorithm, the restriction to examination of only the largest singular values must be relaxed. The comparison between the DORT algorithm and the TFM was then carried out in application to the experimental data arising from the inspection of a lack-of-fusion crack between two welded austenitic plates. Similar discoveries were made; an increased SNR was achieved using the DORT algorithm suggesting that it is more suitable for detection of flaws within noisy media. The position of the crack along the horizontal axis was skewed in the DORT reconstruction, and it was suggested that this could be attributed to its 50° tilt which causes the highest amplitude scattering to be received by elements to the left of the flaw. There was no such problem in the TFM reconstruction and the angled crack was reasonably well characterized. However, a lower SNR of 16.8 dB was achieved. From these scenarios, it is concluded that in its current form, where only the largest singular values are exploited, the DORT method cannot be used successfully for flaw characterization. However, it shows an improved ability over the TFM to separate the flaw scattering from that of the host medium.

## Data accessibility

The research data associated with this paper are available at the following links: http://dx.doi.org/10.15129/086404bd-eb69-429b-978c-2c35cdbfcf87; http://dx.doi.org/10.15129/179e1b38-e701-443d-b995-a4449851330c.
